# Lactase non-persistent genotype influences milk consumption and gastrointestinal symptoms in Northern Russians

**DOI:** 10.1186/1471-230X-11-124

**Published:** 2011-11-13

**Authors:** Yulia Khabarova, Suvi Tornianen, Sari Tuomisto, Irma Järvelä, Pekka Karhunen, Mauri Isokoski, Kari Mattila

**Affiliations:** 1 Department of Family Medicine, Northern State Medical University, Arkhangelsk 163000, Russia and Medical School, Tampere University 33014, Finland; 2 Department of Medical Genetics, University of Helsinki, Helsinki 00014, Finland; 3 Medical School, University of Tampere and Tampere University Hospital 33014, Finland; 4 School of Health Sciences, University of Tampere, Tampere 33014, Finland; 5 Medical School, University of Tampere, 33014 and Center of General Practice, Hospital District of Pirkanmaa, Tampere 33521, Finland

## Abstract

**Background:**

Milk is an important source of nutrients. The consumption of milk, however, may cause abdominal complaints in lactose intolerant individuals. The frequency of -13910C/C genotype is known to be high among Northern Russians, exceeding the prevalence in northern Europe. In our study we tested two hypotheses: 1) subjects with lactase non-persistent genotype (-13910C/C) have more gastrointestinal (GI) symptoms associated with milk 2) subjects with lactase non-persistence avoid using milk.

**Methods:**

In total, 518 students aged 17 to 26 years were randomly selected from different departments in the Northern State Medical University (NSMU) for genotyping the lactase activity-defining -13910C/T variant. All subjects filled in a questionnaire covering their personal data, self-reported GI symptoms and milk consumption habits.

**Results:**

Northern Russians consume very small amounts of milk daily. Among carriers of the lactase non-persistent (LNP) genotype there were 10 percentage units of milk-consumers fewer than among lactase-persistent (LP) subjects (p = 0.03). Complaints of GI disorders caused by milk were different between the genotypes (p = 0.02). Among all types of food analyzed only milk was associated with increased GI symptoms among subjects with the LNP genotype (OR = 1.95, CI 1.03-3.69)

**Conclusions:**

Subjects with -13910C/C have more GI symptoms from milk. Subjects with lactase non-persistent genotype avoid using milk. In the case of increasing milk consumption symptoms may increase the need for medical consultation. It is thus important either for people themselves or for health care staff to be aware of lactase persistence/non-persistence.

## Background

Milk is an important source of everyday nutrition. Bovine milk is a rich resource of nutrients containing lipids, proteins, amino acids, vitamins and minerals. Such substances as immunoglobulins, hormones, growth factors, cytokines, nucleotides, peptides, polyamines, enzymes and other bioactive peptides are also present in milk [1,2]. Milk has been considered a major source of calcium as well as the simplest and most "cost-effective" way to ingest calcium [3,4]. In that dairy products also provide other important nutrients, their consumption improves the nutritional quality of the diet appreciably [4,5].

This notwithstanding, the consumption of milk has decreased over the last decade in Northern Europe [1,5]. Data on milk consumption in Russia in recent years have been very limited and available only from market analyses, which show a decrease in milk consumption in some regions of country [6]. Consumption is low among young Northern Russians [7]. The above-mentioned properties of milk make it important for everyday nutrition, especially for children. Recently a national program, "School milk", aimed to supply schoolchildren with the essential amount of milk daily, has been implemented in Russia [8].

Adult-type hypolactasia, characterized by the down-regulation of lactase enzyme activity in the intestine during human growth, is inherited as an autosomal recessive trait [9,10]. The first genetic variant associated with adult-type hypolactasia, a one-base polymorphism C > T -13910 (rs 4988234) upstream of the lactase encoding gene on chromosome 2, was identified in 2002[11]. This -13910 C/T is the most common variant of lactase persistence among Caucasians [12]. Several other population-specific variants nearby have been identified, some of them only suggestive [13-16]. The -13910C/T has been shown to be located in the OCT-1 binding site and acts as an enhancer. Nowadays the genotyping can be used as the test for adult-type hypolactasia with 100% specificity in cases of unspecific abdominal complaints [17,18].

Milk contains disaccharide lactose, which may cause gastrointestinal problems in adults by reason of its poor digestion in part of population. In Northern Europe lactase persistence is common, which allows the majority of the population to drink milk without any consequences [19]. However, in populations where the frequency of the lactase persistent genotype is rather low the consumption of milk may cause complaints in the digestive tract [20]. The development of symptoms depends on the amount of milk consumed as well as on individual sensitivity. It has been shown that subjects with hypolactasia can tolerate moderate quantities of milk, up to 12 g of lactose/200 ml of milk. If the daily dose of lactose is consumed in small portions and also with a meal, the likelihood of symptoms is low [20-22].

The most common gastrointestinal (GI) symptoms which characterize intolerance to lactose are flatulence, diarrhea, gurgling, abdominal distension and abdominal cramping [22-24]. The appearance of symptoms is clearly explained by mechanism of lactose utilization. Lactase-phlorizin hydrolase splits the entering lactose into two monosaccharides, glucose and galactose. The lack of lactase activity leads to intake of indigested lactose into the bowel. The osmotic effect causes diarrhea [25], while an increased level of fermentation in the intestine leads to increasing production of gases and thus to flatulence [26,27].

The prevalence of adult-type hypolactasia (lactase non-persistence, -13910C/C) among Northern Russians has been found to be 35% [7]. It has also been shown that the prevalence of the -13910C/C genotype among Russians living in the central part of the country ranges from 36 to 50% [28,29]. This frequency is twice as high as in Finland and many times higher compared with other Scandinavian countries [30-33]. It also exceeds the prevalence of the -13910C/C genotype among Estonians [34].

Recent studies have shown that subjects with intolerance to lactose tend to reduce their consumption of milk, which is predictable as far as they suffer from symptoms after milk consumption [31,35-38]. In our previous study [7] subjects showed no differences between genotypes in the frequency of GI complaints during life. We found, however, that the consumption of milk is extremely low among the Northern Russian population compared with other Northern populations. Does milk consumption influence the appearance of GI symptoms in a population with a high frequency of the lactase non-persistent genotype but with very low consumption of milk on the whole?

In the present study we sought to test two hypotheses: 1) subjects with lactase non-persistent genotype (-13910C/C) possibly have more gastrointestinal symptoms associated with milk 2) subjects with lactase non-persistence avoid using milk.

## Methods

### Study population

Students aged 17 to 26 years were randomly selected for this investigation from different departments in the Northern State Medical University (NSMU) during the period from 2006 to 2008. Blood samples (n = 241) or buccal samples (n = 277) were taken from 518 subjects in total for the genotyping of the lactase activity-defining -13910 C/T variant.

The first group were recruited during the period 2006-2007 and blood samples were drawn for the genotyping. The second group were recruited during the period 2007-2008 and buccal samples were taken for the same purpose. The mean age for whole group was 19.8 ± 1.72. Women represented 78.2% of all participants. Both groups of students were similar with respect to distributions of age and gender.

For randomization we used the official list of students of the NSMU. Since there are 16 faculties in the University and each has 10 to 12 groups of students we took for our study every third group from every fourth faculty.

The study was approved by the Ethics Committee of the NSMU (No 08/06 from 29.11.2006). All subjects gave their informed consent to participate.

### Analyses of genotype

Blood samples were genotyped at the Department of Medical Genetics, University of Helsinki. Buccal samples were genotyped at the Forensic Laboratory in the University of Tampere.

Genotyping of blood samples was performed as previously described [11]. DNA was amplified by polymerase chain reaction (PCR). We used Taq polymerase (Dynazyme, Finnzymes, Espoo, Finland) with the conditions described elsewhere. The forward primer used was 5'-CCTCGTTAATACCCACTGACCTA-3' and the reverse primer was 5'-GTCACTTTGATATGATGAGAGCA-3', which cover about 400 bp regions on both sides of the C/T-13910 variant. The PCR product was verified by 1.5% agarose gel electrophoresis (with ethidium bromide). The PCR products were purified using Shrimp Alkaline Phosphatase (USB) and Exonuclease I (New England Biolabs) at 37°C for 60 min and at 80°C for 15 min.

In sequencing, a BigDye 3.1 terminator (Applied Biosystems) was used according to the manufacturer's instructions. Sequencing conditions were as follows: at 96°C for 1 min, then 25 cycles at 96°C for 10 s, at 55°C for 5 s and at 60°C for 4 min. The sequencing reaction followed purification on Millipore Multiscreen plates (Millipore, USA) with Sephadex G-50 Superfine sepharose (Amersham Biosciences, Sweden), electrophoresis by ABI 3730 DNA Analyzer (Applied Biosystems) and base calling by Sequencing Analysis 5.2 software (Applied Biosystems). The sequence obtained was analyzed using Sequencher 4.6. software (Gene Codes, USA).

The polymorphism of lactase persistence/non-persistence SNP rs4988235 from buccal samples was determined by TaqMan Human Custom Genotyping Assay from Applied Biosystems. The assay was performed according to the instructions provided with the assay with an ABI Prism 7900 HT sequence detection system (AppliedBiosystems, California, USA).

### Questionnaire

All subjects gave written informed consent and filled in a questionnaire covering their personal data, self-reported condition of health, GI symptoms and milk consumption habits. We used the questionnaire with permission from Sahi [10].

Gastrointestinal symptoms were estimated by the question "Have you ever experienced the following symptoms and in what frequency?" The question was not intended to clarify the connection between milk consumption and symptoms. The various answers were "Every day", "At least once per week", "Every second week", "Sometimes". If the participant had never had the symptom the item was left blank. We classified the answer "Sometimes" together with no symptoms into one group and marked it "Without GI symptoms". The second group included the remaining variables and was taken for analyses of differences between genotypes.

The influence of food on the appearance of GI disorders was estimated by the question "Do you have any GI disorders if you consume the following types of food?" where six types of food being included. Subjects were asked to put "Yes" or "No" opposite every type of food.

In the last part of the questionnaire subjects were asked to define their everyday milk product consumption from 0 to more than 5 glasses per day. The choices were "Not at all", "1-2 glasses", "3-5 glasses" and "More than 5 glasses" of milk/sour milk per day. There was also an option to declare consumption less than 1-2 glasses per day for those who consumed milk very rarely. For the analysis we classified all answers into two groups: less than daily milk consumption or none and daily milk consumption.

Comparison was made between the lactase non-persistent group (-13910C/C) and the lactase persistent group (-13910C/T and -13910 T/T) for all the above-mentioned questions.

### Statistical analysis

The difference between genotype groups in milk consumption and others were tested by χ2 test. Odds ratios (OR) with 95% confidence interval (CI) for -13910C/C genotype in the logistic regression analysis were calculated for gastrointestinal symptoms in total and symptoms after several types of food, and for volume of milk/sour milk consumption. Statistical analyses were performed with SPSS version 15.0 (SPSS Inc. Chicago, IL, USA).

## Results

The prevalence of the -13910C/C genotype (lactase non-persistence, LNP) among 518 young students from North-West Russia was found to be 35% (n = 180) while the -13910C/T genotype was found in 46% and -13910T/T in 19% of participants.

About sixty percent of all subjects reported GI symptoms (Table 1). The most frequent symptom was stomach-ache. However, no statistically significant differences were revealed in the frequency of gastrointestinal symptoms in total between the LP and LNP genotype groups. The study brought out no statistically significant differences in symptom appearance between male and female participants.

**Table 1:** The frequency of gastrointestinal symptoms (GI) among young people with lactase-persistent/non-persistent genotype (Question "Have you ever experienced the following symptoms?")

	Lactase non-persistence C/C-13910 (n = 180)		Lactase persistence C/T and T/T-13910 (n = 338)		
	
GI symptoms	n	%	n	%	p
Stomach-ache	93	51.7	176	51.9	1.00

Regurgitation	23	12.8	42	12.4	0.89

Flatulence	17	9.4	38	11.2	0.65

Heartburn	14	7.8	23	6.8	0.72

Nausea	13	7.2	25	7.4	1.00

Diarrhea	3	1.7	12	3.5	0.28

Without GI symptoms	74	41.1	142	42.2	0.71

The majority of subjects consumed very small amounts of milk daily. Only about 40% of all participants used milk every day. Almost 50% did not use milk at all and 13% reported consumption of milk more seldom than daily (Table 2). Among carriers of the LNP genotype there were 10 percentage units fewer milk-consumers in comparison with the LP group (32.2 and 42.0%). The differences were statistically significant (p = 0.03). Sour milk consumption did not show significant differences in symptoms between LP and LNP subjects.

**Table 2:** Milk consumption by genotype

Consumption of milk	Lactase non-persistence -13910C/C (n = 180)		Lactase persistence -13910C/T and -13910T/T (n = 338)	
	
	n	%	n	%
Less than daily or none	122	67.8	196	58.0

Daily	58	32.2	142	42.0

Total	180	100	338	100

Milk consumption was associated with GI disorders in 13.3% of subjects with the LNP genotype and in 7.1% of subjects with the LP genotype (Table 3). Complaints of GI disorders caused by milk consumption were different between the genotypes (p = 0.02).

**Table 3:** The frequency of GI disorders connected to different food stuffs (Question "Do you have any GI disorders if you consume the following types of food?")

Type of food	Lactase non-persistence -13910C/C (n = 180)		Lactase persistence -13910C/T and -13910T/T (n = 338)		p
		
	n	%	n	%	
Fatty	56	31.1	120	35.5	0.33

Fried	43	23.9	69	20.4	0.37

Milk	24	13.3	24	7.1	0.02

Sour milk and kefir	16	8.9	17	5.0	0.09

Vegetables	10	5.6	14	4.1	0.51

Fruits	7	3.9	19	5.6	0.53

Any other food	28	15.6	52	15.4	1.00

Not any food	88	47.6	174	51.6	0.74

Among all types of food analyzed only milk resulted in statistically significant differences in symptoms between LP and LNP subjects (OR = 1.95, CI 1.03-3.69) (Table 4). In regression analysis there was no connection between consuming food and the appearance of symptoms except in milk consumption.

**Table 4:** Odds ratios (OR, 95% CI) of the lactase non-persistent genotype (-13910C/C) in the logistic regression analysis for gastrointestinal symptoms, symptoms after some food and milk consumption.

GI symptoms	OR (95% CI)
Diarrhea	2.12 (0.58 - 7.76)

Flatulence	1.17 (0.62 - 2.21)

Nausea	1.04 (0.50 - 2.14)

Stomach pain	1.00 (0.68 - 1.46)

Regurgitation	0.98 (0.53 - 1.76)

Heartburn	0.82 (0.39 - 1.71)

Symptoms after some food	

Milk	**1.95 (1.03 - 3.69**)

Sour milk and kefir	1.61 (0.75 - 3.43)

Fried	1.61 (0.97 - 2.67)

Vegetables	1.25 (0.52 - 3.00)

Any other food	1.02 (0.59 - 1.75)

Fatty	0.68 (0.43 - 1.07)

Fruits	0.52 (0.20 - 1.31)

Milk product consumption	

Milk	**1.51 (1.04 - 2.17**)

Sour milk	1.10 (0.76 - 1.60)

Statistically significant differences are presented in bold face.

## Discussion

Young Northern Russians consume very small amounts of milk daily. However, among LNP genotype carriers there are even less milk-consumers than among LP subjects. Our findings demonstrate that milk is the only type of food having an influence upon the appearance of GI disorders differently by genotype. We may conclude that subjects with lactase non-persistence (-13910C/C) have more symptoms from milk. Subjects with lactase non-persistent genotype avoid using milk.

Sour milk consumption brought out no differences between the genotypes. This is in accord with the previous proved finding that sour milk is better tolerated than milk by persons with lactose intolerance [21,39]. Sour milk contains less lactose as a result of bacterial fermentation.

All students recruited for this study were considered healthy. However, only forty per cent of them did not report any GI symptoms and half of them reported stomach-ache, which was the most frequent symptom among all subjects without links to genotype. Possibly this finding requires further investigation of student health to clarify the causes of the frequent GI disorders among a healthy young population.

Regarding other populations it was shown that only flatulence occurs significantly more frequently among LNP subjects [39,40]. Jussila and associates [41] showed that loose stool was a more frequent symptom in subjects with selective lactose malabsorption.

The northern Russians use very small amount of milk daily. However LNP subjects use less milk than people with LP genotype. Such a self-prescribed reduction is in agreement with a study in Finnish children with the -13910C/C genotype, who naturally diminished their usage of milk [31] and also with a study in children of other ethnicities [42]. It was also shown that other age groups also tend to reduce their milk consumption because of the appearance of symptoms after milk [24,38].

It is possible to assume that the low rate of symptoms was partly related to the low milk intake in our study population. It is likely even if the prevalence of adult type hypolactasia in the study population is as much as 35% and low milk consumption is reported symptoms appear less probably than in other populations. Another explanation for the low rate of symptoms may be the age of lactase activity down-regulation. Since there are no studies estimating the age of decline of enzymatic activity in Russians there is a slight possibility that this group was too young to demonstrate differences in GI disorders.

Vast majority of our randomly selected students were from North-West Russia [7]. Based on the farming history, this area was determined in Soviet Union as milk production area. The region has still been considered as one of the country's milk and meat production areas. The gene-culture co-evolution between cattle milk genes and humans lactose tolerance has been verified for North Central Europe [43] and it seems to be also in north-western part of Russia.

Since milk is an important source of nutrients there are numerous studies aimed to demonstrate the advantages and disadvantages to milk restriction or elimination from the diet. The lack of milk in nutrition in the young population is strongly associated with decreased bone mineral density and leads to osteoporosis in older age [42]. Precise statistic data of prevalence of osteoporosis among the Russian population are lacking. However, it is obvious that osteoporosis as well as a higher risk of bone fractures connected with it is an actual problem for Russia [44]. Building peak bone mass during childhood and adolescent can be the best means of preventing osteoporosis in older age.

## Conclusions

It is surely warranted to increase milk consumption among the Russian population by providing a federal program. The benefits from an increase in milk consumption are obvious. At the same time our study has shown that lactase non-persistent subjects have higher frequency of GI disorders even in population with very small amount of milk consumed. It may be predicted that in the case of increasing milk consumption symptoms may appear more frequently and may increase the need for medical consultation and care. It is therefore important either for people themselves or for health care staff to be alert to lactase persistence/non-persistence.

## Competing interests

The authors declare that they have no competing interests. This research received no specific grant from any funding agency in the public, commercial or not-profit sectors.

## Authors' contributions

YK designed the research, collected the material, processed the data, and drafted the manuscript. ST made the genotyping of blood samples. ST made the genotyping of buccal samples and drafted the manuscript. IJ designed the research, supervised the research, and helped to draft the manuscript. PK supervised of analyses of genotypes (buccal samples), and help to draft the manuscript. MI proposed the study idea, supervised, supported and designed the study, and drafted the manuscript. KM supervised, supported and designed the study, supervised data processing, and drafted the manuscript.

All authors read and approved the final manuscript.

## Pre-publication history

The pre-publication history for this paper can be accessed here:

http://www.biomedcentral.com/1471-230X/11/124/prepub
